# Personalized quantification of facial normality: a machine learning approach

**DOI:** 10.1038/s41598-020-78180-x

**Published:** 2020-12-07

**Authors:** Osman Boyaci, Erchin Serpedin, Mitchell A. Stotland

**Affiliations:** 1 Electrical and Computer Engineering Department, Texas A&M University, College Station, 77843 USA; 2 Division of Plastic and Craniofacial Surgery, Department of Surgery, Sidra Medicine, C1-121 OPC, Doha, 26999 Qatar; 3 Department of Surgery, Weill Cornell Medical College-Qatar, Doha, 26999 Qatar

**Keywords:** Health care, Medical research, Engineering, Mathematics and computing

## Abstract

What is a *normal* face? A fundamental task for the facial reconstructive surgeon is to answer that question as it pertains to any given individual. Accordingly, it would be important to be able to place the facial appearance of a patient with congenital or acquired deformity numerically along their *own* continuum of normality, and to measure any surgical changes against such a personalized benchmark. This has not previously been possible. We have solved this problem by designing a computerized model that produces realistic, normalized versions of any given facial image, and objectively measures the perceptual distance between the raw and normalized facial image pair. The model is able to faithfully predict human scoring of facial normality. We believe this work represents a paradigm shift in the assessment of the human face, holding great promise for development as an objective tool for surgical planning, patient education, and as a means for clinical outcome measurement.

## Introduction

The human face is an indispensable, dynamic organ of social communication. Even excluding the rich signaling information provided by animative expression, a cursory glance at a face instantaneously prompts an array of subconscious interpretation within an observer. Consequently, the social implications of congenital and acquired forms of facial disfigurement on affected individuals are significant, and are underscored by the widespread desire of patients to seek normalizing interventions. For the facial reconstructive surgeon, the primary task during clinical assessment and planning is to determine what *normal* actually means as it pertains to any given individual face. Previous related research, though wide-ranging, has bypassed the issue of objectively customizing facial analysis, while considering matters such as the global determinants of beauty (e.g., proportion, symmetry, averageness)^1–5^, communication and perception of emotion^6,7^, personality inference^8,9^, morphing techniques^10^, regions of attraction^11–14^, machine recognition^15^, and computation of anthropometric and digital population norms^16^. Various methods of facial assessment introduced over the past decades, including expert ratings^17,18^, anthropometric landmark measurements^19^, stereophotogrammetry studies^20^, crowdsourced surveys^21^, patient-reported outcomes^22,23^, and eye-tracking analyses^14,24^, are not benchmarked against a given patient’s own theoretical facial norm (nor do they lend themselves easily to application in the clinical setting). Further, despite the fact that population means for facial appearance can be determined using large database averaging techniques, it is important that reconstructive interventions be carried out within the context of a patient’s own unique, anatomic features. This is because the distinctive facial features of any given patient—influenced by gender, age, race, etc.—are not likely to be reflected accurately by a calculated norm derived from the broader population. Attempting to address this issue by finely segmenting massive databases into increasingly narrow demographic classifications would be confounded by the historical admixture of human populations and a globalized world with increasingly mixed lineage. That is, building a library of facial norms to provide a credible reference source that faithfully matches the multidimensional singularity of any given patient seems unrealistic.

Beyond the importance of being able to define patient-specific normality, two particular measures currently unavailable would be beneficial assets for a treating clinician: (1) a means of placing an individual’s facial appearance numerically along their *own* hypothetical continuum of normality, and (2) an objective, reproducible method to quantify the change effected by any reconstructive intervention. We have designed a novel solution to address these gaps by constructing the first computerized model capable of automatically producing realistic, normalized versions of any given face. Our approach melds raw (i.e., real) images of individuals, with images created by an open-source generative adversarial network (the “Style GAN”)^25^. This GAN was designed using a 70,000-image database that is broadly distributed across gender, age, and ethnicity. Our model also employs a Learned Perceptual Image Patch Similarity (LPIPS) function^26^ and the method of stochastic gradient descent (SGD) to generate a new facial image that integrates elements of any inputted image along with components derived from the GAN database. The system has been calibrated in such a way as to naturally correct facial deformities by an iterative, multi-objective optimization procedure within the latent space of the GAN. Moreover, we devised a method to measure the difference between any two images in image space via perceptual similarity (LPIPS)^26^, structural similarity (mean structural similarity index measure—MSSIM)^27^ and peak signal-to-noise ratio (PSNR) techniques^28^, as well as in latent space using Bray–Curtis distance^29^ and correlation distance measures. Following these methods of feature extraction, we obtained human ratings of two distinct sets of facial images, then used the Multi-Layer Perceptron (MLP) neural network regressor to train a prediction model on one set, and to test its accuracy on the second set.

It should be noted that all prior references in the scientific literature to concepts akin to “face normalization” apply that term differently than we do here, relating it instead to automatic adjustments of image alignment, illumination, and pose. These systems routinely pertain to facial recognition tasks which require canonical facial views to augment the model’s performance^30–33^, or to processing steps used to improve consistency and reliability of clinical image analysis^34^. Two recent reports describe the use of GANs for ophthalmological assessment. The first employs a GAN in an unsupervised manner to learn a diversity of normal anatomical variability within optical coherence tomography (OCT) images of the retina^35^. They developed a model simply to label anomalies within the OCT images, based on measured deviations from the learned distribution; no image normalization was performed. In another study focusing on thyroid-associated ophthalmopathy, a GAN was trained on pairs of matched pre-and postoperative facial images^36^. The objective of this study was to synthesize a realistic facsimile of post-operative periorbital appearance in order to manage the expectations of prospective surgical patients. Only 109 image pairs were used to train the GAN, the image resolution was poor ($64 \times 64$), and they were thus unable to preserve the unique features of the facial images, which they acknowledged were “limited by low realism”. Moreover, no effort was made in that study to use machine learning techniques to measure distance between pre- and post-operative images. In fact, to the best of our knowledge, there is no precedent in the literature prior to our study for a machine learning system designed to realistically normalize facial images by eliminating deformity, nor to measure the generated variance between raw and normalized images.

We believe that this work represents a paradigm shift in the assessment of the human face, and holds great promise for development as a vital tool for surgical planning, patient education, and as an innovative means for clinical outcome measurement.

## Results

**Figure 1:**
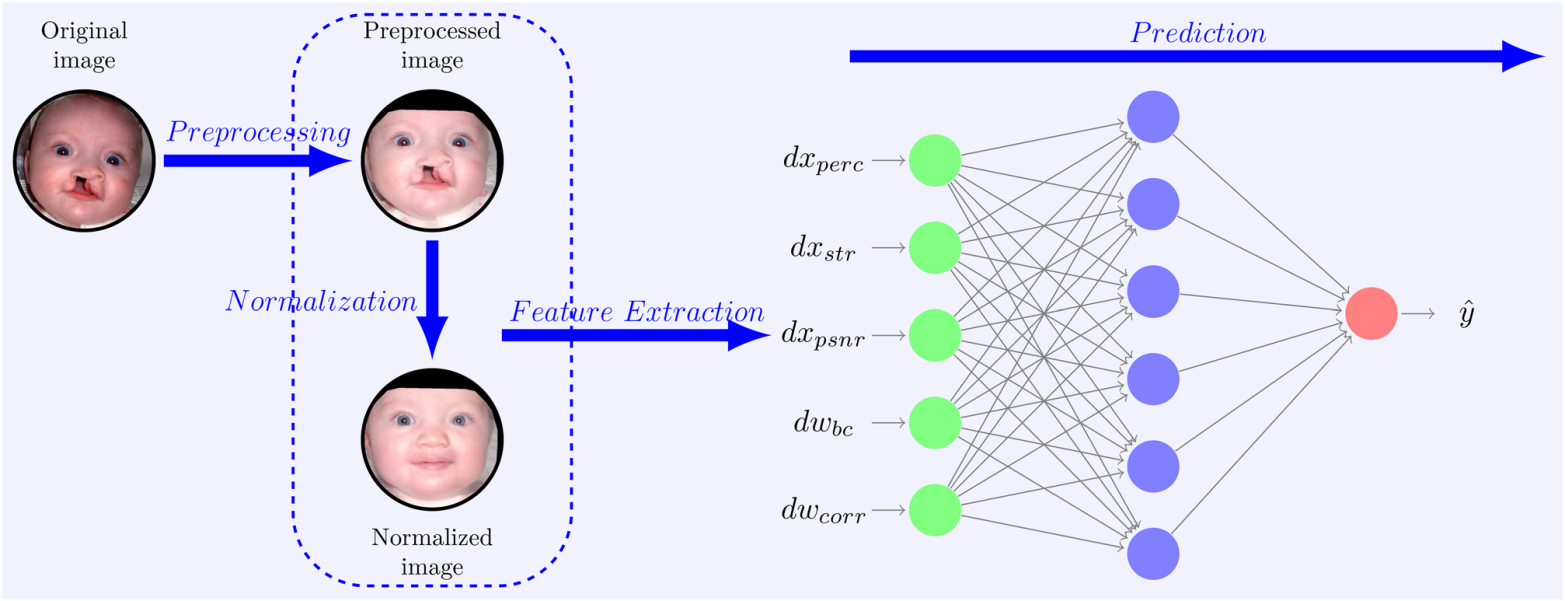
General signal flow and high level architecture of the study model demonstrating the four key steps: preprocessing, normalization, feature extraction, and prediction. As a first step, preprocessing is applied to the original image to obtain a cropped, aligned and masked image. Then, the preprocessed image is normalized by preserving its unique characteristics and filtering out any structural anomalies. As a next step, features are extracted by measuring the distances between normalized and preprocessed images and latents both in the image space and in the latent space. While the features $dx_{perc}$, $dx_{str}$, and $dx_{psnr}$ denote the perceptual distance, structural distance, and PSNR distance in image space, $dw_{bc}$ and $dw_{corr}$ denote Bray–Curtis distance and correlation distance in latent space, respectively. After retrieving the representative features, human rating is predicted (${\hat{y}}$) using the MLP regressor with only one hidden layer having 6 neurons. Original image, reference^37^.

An overview of our study model is depicted in Fig. 1. The proposed computational model consists of four steps:

1. Image preprocessing.
2. Image normalization.
3. Image feature extraction.
4. Prediction of image scores.

### Image preprocessing

Preprocessing was the first step of our model in which a face within an image was detected, centered, cropped, aligned, and masked so that the main components of the face were located in a predetermined orientation^25,38,39^. We labeled the output of this preprocessing phase $x_{raw}$.

### Image normalization

The second step of our model was normalization, in which anomalous elements in $x_{raw}$ following the image preprocessing step were corrected without altering the “normal” features of the face. The output of this step was labeled $x_{nrm}$.

**Figure 2:**
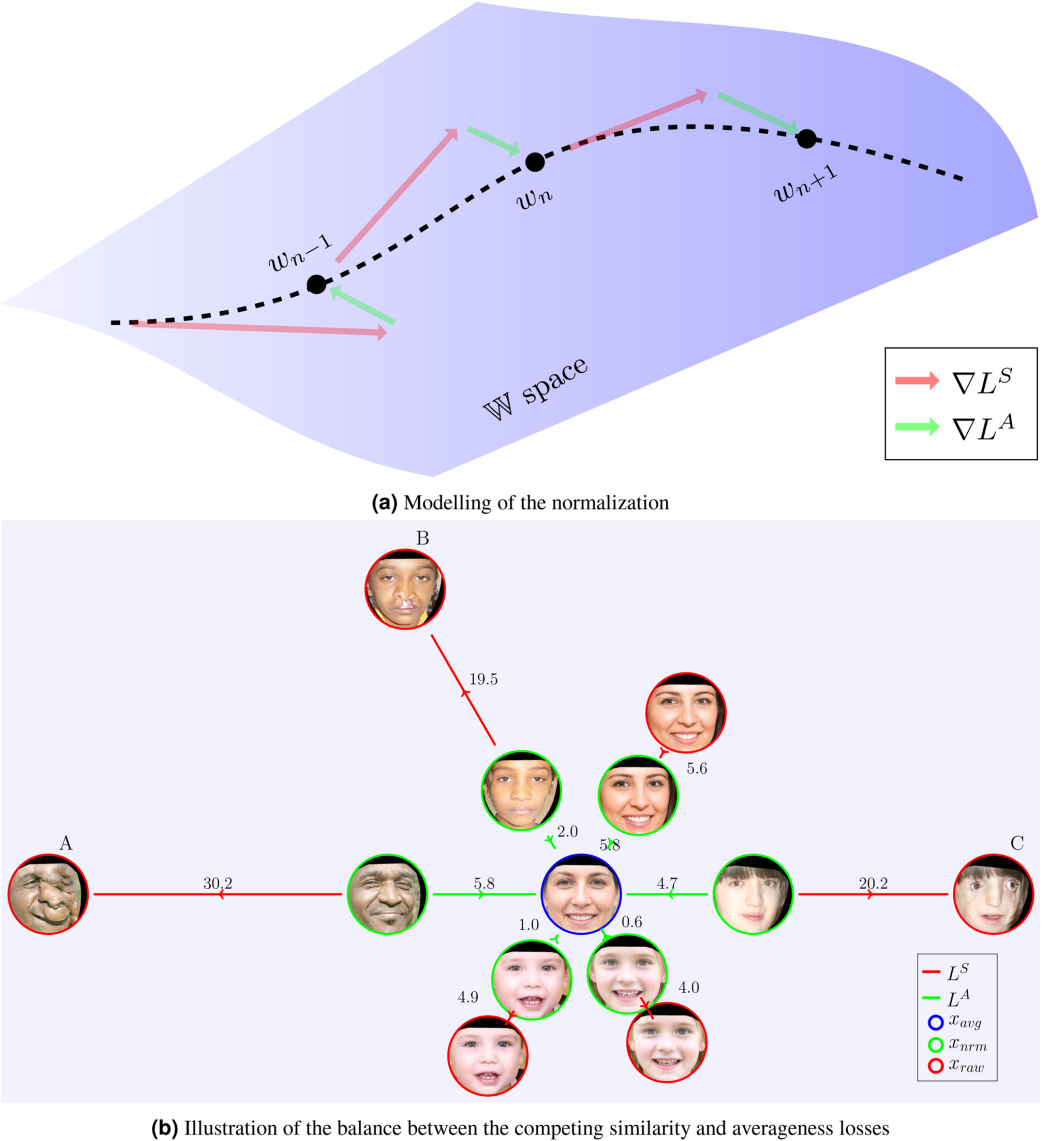
Visualization of the optimization represented by Eq. (1). In (**a**) modeling of the iterative normalization procedure in latent space ${\mathbb {W}}$ is depicted. The dashed curved line represents the “normal” manifold in ${\mathbb {W}}$, and $w^*$ is found along that curve by iterative updates. At each iteration *i*, the next latent vector $w_{i+1}$ is found by adding the gradient vector of similarity loss $\nabla L^{S}$ and the gradient vector of averageness loss $\nabla L^{A}$ to the current latent vector *w*. Note that $L^{S}$ is calculated in image space ${\mathbb {X}}$, and gradients are back-propagated to the *w*. In contrast, $L^{A}$ is calculated in the ${\mathbb {W}}$ space, and thus gradients are calculated directly. These two conflicting gradients were calibrated in a manner that $w^*$ satisfies both similarity and averageness objectives upon completion of the normalization operation. In (**b**) trade-off between image similarity loss ($L^S$) and latent averageness loss ($L^A$) is illustrated. We obtained $x_{nrm}$ (green) for each $x_{raw}$ (red) using our normalization algorithm. The distances between the images separated by red lines reflect the distance in image space between $x_{raw}$ and $x_{nrm}$ measured with $L^{S}_{perc}$. Similarly, the distances between the images separated by green lines reflect the distance in latent space between $w_{raw}$ and $w_{avg}$ measured with $L^{A}_{mse}$. Note that while $L^{S}$ attempts to produce images *similar* to $x_{raw}$, the objective of $L^{A}$ is to converge upon the population *average*. All numbers above are multiplied by 100 for better visualization. Images A,B,C, above, correspond to references^40–42^, respectively. Images within red and blue circles, above, derive from the StyleGAN face generator^25^. All faces within green circles, above, are transformations created by the study encoder.

We formalized the normalization procedure as a two-objective optimization problem, considering both *similarity* and *averageness* losses. While the objective of similarity loss was to preserve the distinctive elements of the face, averageness loss drove the correction of any structural aberrancies. Optimization was carried out within the latent space ${\mathbb {W}}$ of the StyleGAN’s generator *G* in order to find a latent vector $w^*$ in ${\mathbb {W}}$, which yielded the best results in terms of similarity and averageness. We represented the similarity loss by $L^{S}$, and the averageness loss by $L^{A}$. The normalization procedure is formally represented by the optimization problem:

1 $$\begin{aligned} w^* = \min _{w} \big (\lambda _{sim} \cdot L^{S}(x_{raw}, G(w)) + \lambda _{avg} \cdot L^{A}(w, w_{avg}) \big ), \end{aligned}$$

where $\lambda _{sim}$ and $\lambda _{avg}$ are weighting constants that we manipulated in order to perform an optimal trade-off balance between the relative importance of the similarity and averageness losses, and $w_{avg}$ denotes the latent vector of the population average. This optimization is represented schematically in Fig. 2a,b.

**Figure 3:**
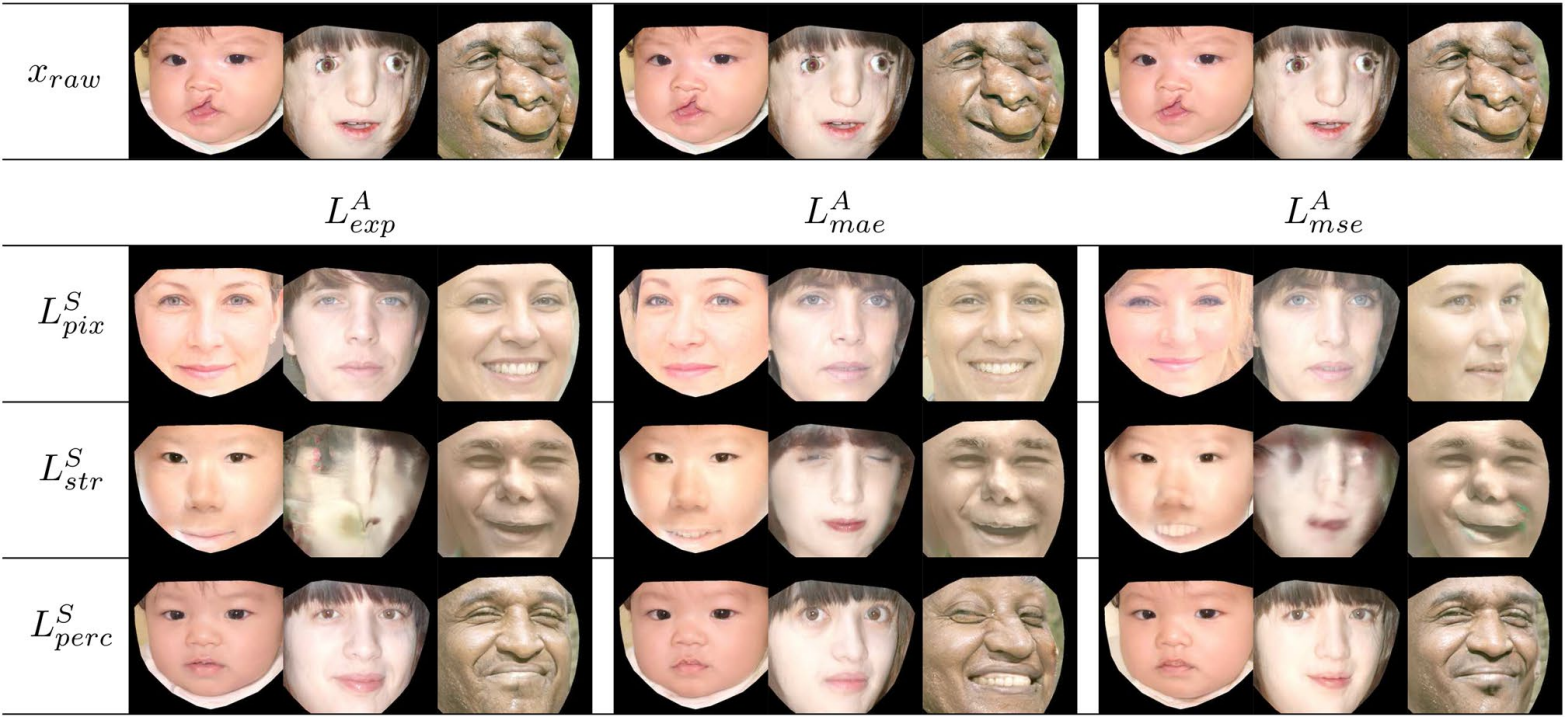
Effects of $L^{S}$ and $L^{A}$ in the normalization procedure. We randomly chose three different raw images in order to run our normalization algorithm to test three different $L^{S}$ and three $L^{A}$ functions. The top row represents the raw images as a reference. The remaining matrix of images below demonstrates the effect of the different similarity ($L^S$) and averageness loss ($L^A$) tools. By examining this matrix it is visually apparent that the optimal combination of $L^S$ and $L^A$ derives from the simultaneous use of $L^S_{perc}$ and $L^A_{mse}$. The three images depicted in the top row, left to right, correspond to references^40,42,43^, respectively. All remaining images are transformations created by the study encoder.

Because the success of the normalization operation depended primarily on filtering out the anomalous features of any given image, while preserving the normal elements, proper selection of $L^{S}$ and $L^{A}$ played a vital role in generating a realistic $x_{nrm}$ from a given $x_{raw}$. In increasing order of algorithmic complexity, we tested the following three methods for $L^{S}$: pixel loss ($L^{S}_{pix}$), structural loss ($L^{S}_{str}$)^27^ and perceptual loss ($L^{S}_{perc}$)^26^. In similar fashion, to measure $L^{A}$ we tested mean absolute error ($L^{A}_{mae}$), mean squared error ($L^{A}_{mse}$), and mean exponential error $L^{A}_{mee}$. Taken together in various combinations, these six methods of loss measurement provide nine different options for normalization. In addition, an unlimited amount of fine tuning of the system was available by manipulation of $\lambda _{sim}$ and $\lambda _{avg}$. For each of the nine combinations of loss functions, we adjusted the $\lambda$ parameters so as to generate the best normalized output possible as determined by investigators’ visual appraisal (Fig. 3). Using this approach, $L^{S}_{pix}$ was found to generate realistic human images, but we noted that certain basic facial features such as gender, age, and pose were misaligned. Similarly, $L^{S}_{str}$, generated relatively blurry images. Conversely, $L^{S}_{perc}$ produced highly realistic images without degrading the main features of a given face. Having determined the optimal $L^{S}$, we then examined the effects of the three different $L^{A}$ on the normalization process and observed that $L^{A}_{mse}$ provided the best result.

Another factor critical to the normalization operation was the initial value of the latent vector *w*, which we assigned empirically. Due to the fact that ${\mathbb {W}}$ is multi-dimensional in nature, simply choosing a random *w* may not be advantageous because the initial value may fall very far from—and never converge towards—$x_{nrm}$. Therefore, we chose $w_{avg}$ as our initial assignment for *w*, and commenced the iteration process from that point. The number of iterations was fixed at 500 since it was observed that no improvement was achieved beyond that point.

**Figure 4:**
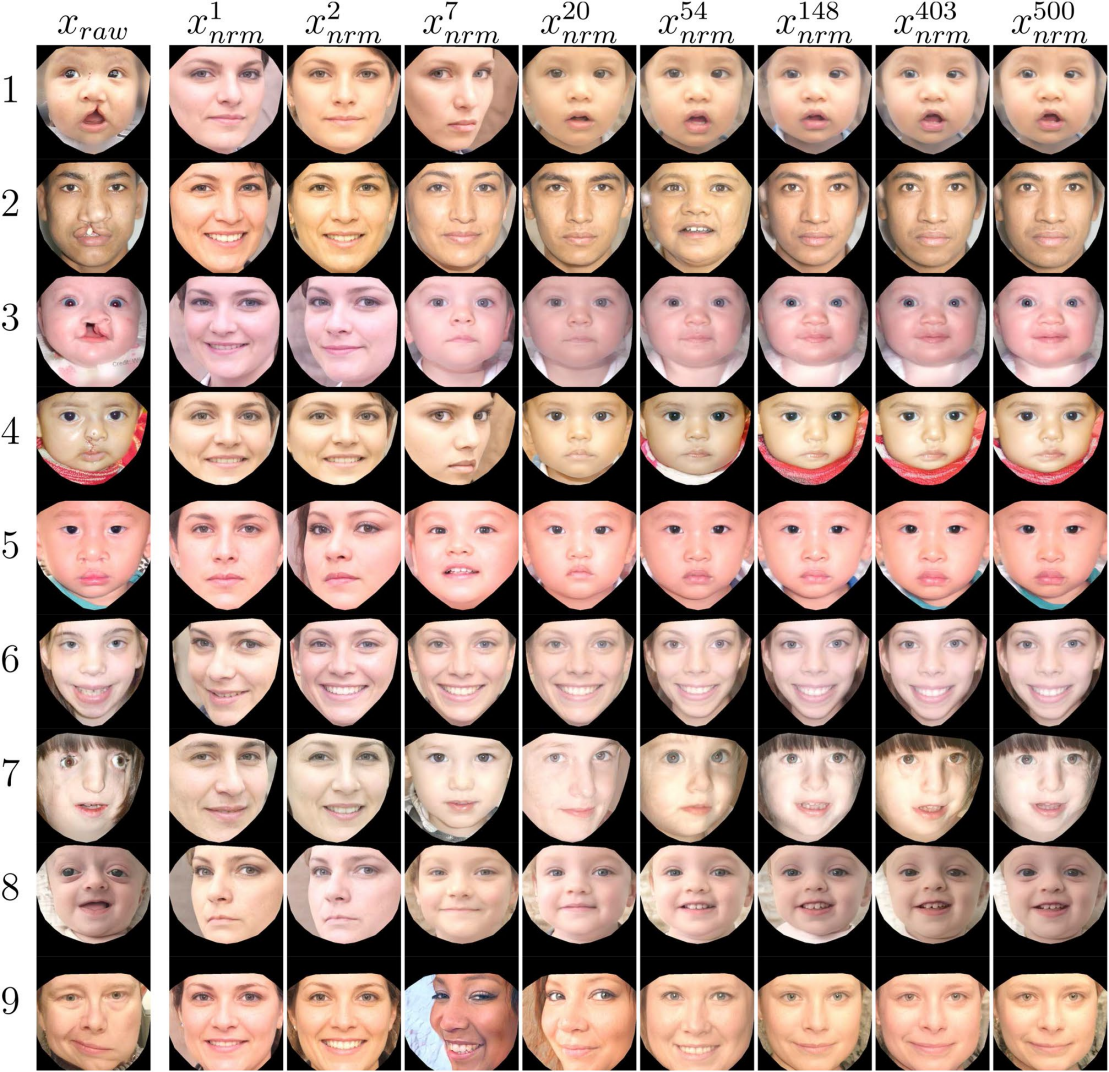
Iterative progression of our normalization technique. Each row represents the progression of an individual image towards its “normalized” version. The first column is $x_{raw}$. Subsequent 7 columns denote the iterations rounded to the nearest integer natural logarithm ($\ln (i)$) for each iteration *i* between 0 and 500, respectively. The final column represents the optimal version out of 500 iterations, namely $x_{nrm}$. References for images in column $x_{raw}$: row 1^44^, row 2^45^, row 4^46^, row 5^47^, row 6^48^, row 8^49^, row 9^50^. All other images are duplicates or transformations created by the study encoder.

**Figure 5:**
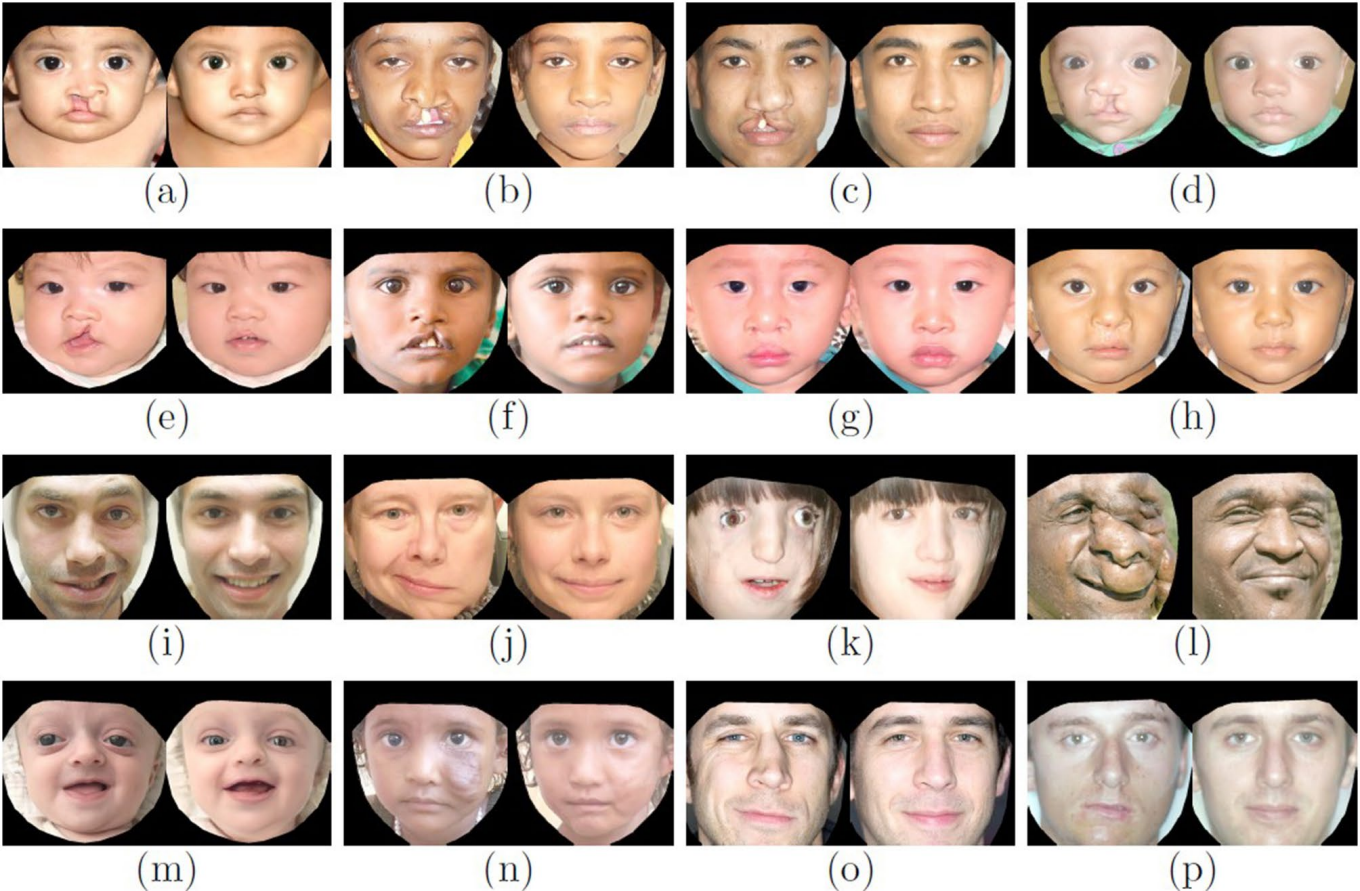
Raw-normalized image pairs for a variety of different clinical diagnostic categories (**a**–**d**) complete unilateral cleft lip; (**e**,**f**) incomplete unilateral cleft lip; (**g**,**h**) reconstructed bilateral cleft lip; (**i**,**j**) unilateral facial palsy; (**k**) Treacher Collins syndrome; (**l**) neurofibromatosis; (**m**) craniofacial dysostosis; (**n**) vascular anomaly; (**o**) nasal deformity; (**p**) jaw asymmetry]. Image references: (**a**)^46^, **d**^51^, (**f**)^52^, (**h**)^53^, **i**^54^, (**n**)^55^, (**o**)^56^, **p**^57^. All remaining images are duplicates or transformations created by the study encoder.

In Fig. 4, the iterative progression of our normalization procedure is demonstrated for nine raw facial images depicting individuals with: incomplete cleft lip, complete cleft lip, craniofacial microsomia, Treacher Collins syndrome, craniofacial dysostosis, and left facial palsy. For purposes of concision, only representative steps are shown (rounded integer iterations of $\ln (i)$ for $i=1-500$). Notice that at the first iteration of normalization, basic features of the given images such as skin color and pose are set. Then as the iterative process continues, secondary features such as age and gender become aligned. Ultimately, the more distinctive elements of the face are seen to emerge. It is critical to ensure that the latent vector *w* always resides along the “normal” manifold within the latent space, and does not stray off which could derail the normalization process. Such a condition was controlled within our algorithm by restricting each member of $w_i$ between $-1$ and 1. If during SGD $w_i$ escaped that interval, it was replaced by a random number generated within that range. In Fig. 5, 16 representative pairs of $x_{raw}$ and $x_{nrm}$ are illustrated. It can be appreciated that the normalization process effectively remedied the abnormal features of the faces, while strictly preserving all key remaining details.

### Image feature extraction

Because the objective of our study was to accurately predict the extent of abnormality within a facial image, it was necessary to extract salient facial features in both the image and latent spaces in an effective manner. In order to achieve this within the image space ${\mathbb {X}}$, distances were measured by employing various techniques including LPIPS ($dx_{perc}$), MSSIM ($dx_{str}$), PSNR ($dx_{psnr}$), mean absolute error ($dx_{mae}$), mean squared error ($dx_{mse}$), root mean squared error ($dx_{rmse}$), and log hyperbolic cosine ($dx_{lcosh}$).

The path to ascertaining distance measures within the latent space was more challenging. While the normalization procedure yielded the latent vector $w_{nrm}$, it did not yield $w_{raw}$. To obtain the latter, we repeated the normalization procedure for each raw image by setting $\lambda _{avg}=0$ to find $w_{raw}$. That is, $L^{A}$ was discarded, and only $L^{S}$ was used in the normalization operation when implemented to determine $w_{raw}$.

**Figure 6:**
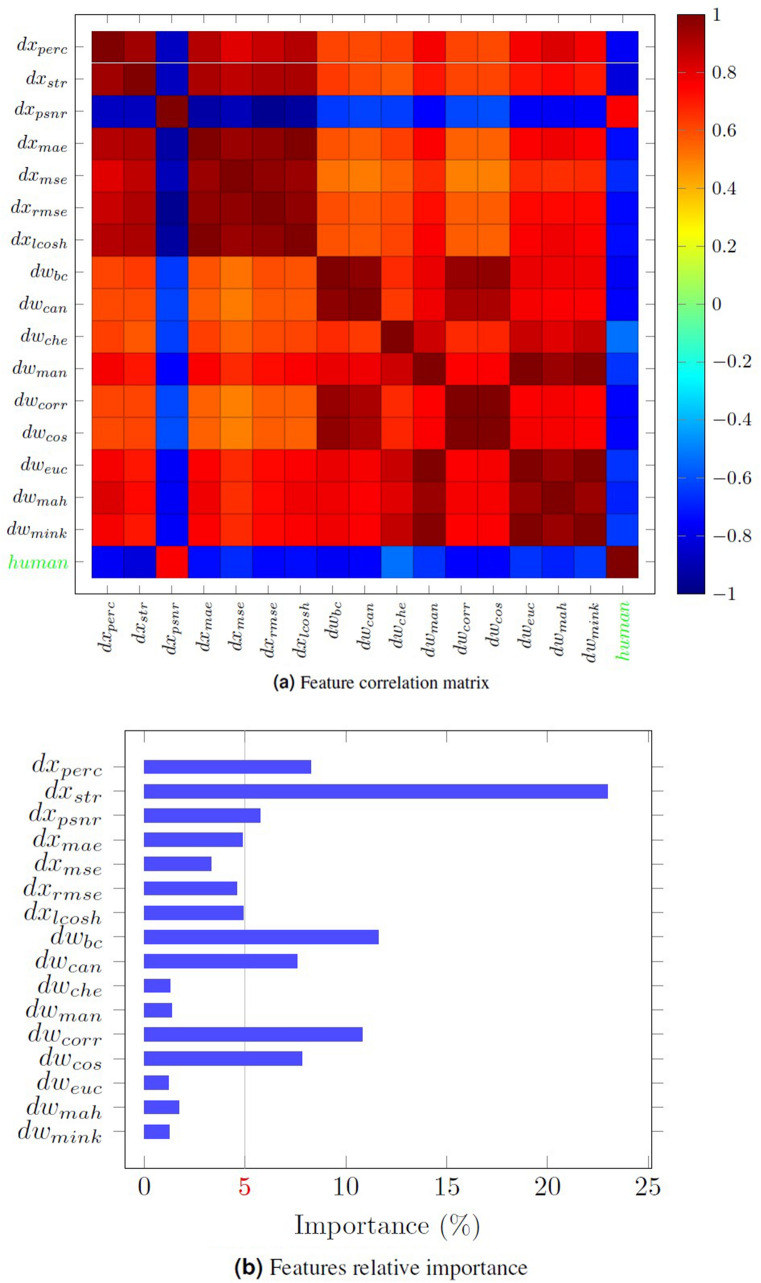
Assessment of extracted features. (**a**) is a matrix demonstrating the correlation between seven different image distance measures, nine different latent distance measures, and human image ratings. (**b**) shows the relative importance of the 16 extracted features using ERT algorithm (see Methods).

After obtaining both $w_{nrm}$ and $w_{raw}$, distances between $w_{raw}$ and $w_{nrm}$ were computed using various distance measures including Bray–Curtis^29^ ($dw_{bc}$), Canberra^58^ ($dw_{can}$), Chebyshev^59^ ($dw_{che}$), Manhattan^60^ ($dw_{man}$), correlation ($dw_{corr}$), cosine ($dw_{cos}$), Euclidean ($dw_{euc}$), Mahalanobis^61^ ($dw_{mah}$), and Minkowski^62^ ($dw_{mink}$). In order to verify the suitability of our selected distance measurements, we tested them against human facial ratings on a training dataset. Figure 6a shows a correlation matrix representing the correlation between our extracted features (first 7 in ${\mathbb {X}}$, and last 9 in ${\mathbb {W}}$), and the correlation between those same 16 features and the human ratings that we collected. Because PSNR measures the similarity between images, $dx_{psnr}$ predictably correlated in a positive manner with the human ratings. Conversely, other features measuring dissimilarity were found to correlate negatively.

As a next step, we applied the extremely randomized trees algorithm (ERT)^63^ to our training dataset to select the best representative features out of those 16. Similar to the random forests algorithm^64^, ERT is an ensemble learning technique which reduces overfitting and improves efficiency of the model by producing a multitude of individual decision trees and averages them to obtain a final prediction. We fitted 1000 randomized decision trees on various sub-samples of the training data in an effort to enhance the prediction accuracy and reduce the over-fitting by using ERT. The relative importance of the features used to predict the human scores can be seen in Fig. 6b. We set the relative importance threshold at 5% as a minimum criterion to include a feature in our model. Therefore, we selected $dx_{perc}$, $dx_{str}$, $dx_{psnr}$, $dw_{bc}$, $dw_{can}$, $dw_{corr}$, and $dw_{cos}$ as our critical features of interest. We eliminated $dw_{can}$ and $dw_{cos}$ since they were so strongly correlated with $dw_{bc}$ (0.99) and $dw_{corr}$ (0.98), respectively. This yielded 5 key predictive features that we used for the remainder of our analysis.

### Prediction of image scores

The final step in developing our facial normality assessment model was to establish its ability to reliably predict human ratings. In order to achieve this, we tested a number of regressors including Linear, Huber, Support Vector, Ridge, Lasso, and Multi Layer Perceptron (MLP) Regressor models. As part of our protocol, we collected scores ($1=\text {abnormal}$; $7=\text {normal}$) from 80 human raters (average age $= 33.96$, 60% male) on 150 private facial images for the purpose of training our regression models. In addition, 50 raters (average age $= 27.5$, 46% male) also scored a separate group of 60 open-source images in order to test the efficacy of our system. Hyper parameters of each regression model were optimized by training on 80% of the training data, and our models were validated on the remaining 20% percent of the training data. Each optimized regression model was then tested on our previously unseen public test data.

**Figure 7:**
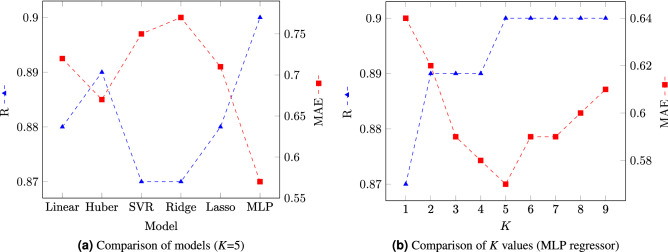
Pearson correlation (R) and mean absolute error (MAE) of the various regression models tested. In (**a**), six regression models including Linear Regressor, Huber Regressor, Support Vector Regressor, Ridge Regressor, Lasso Regressor, and Multi Layer Perceptron (MLP) are compared when $K=5$. MLP yielded the best results: R (0.9) and MAE (0.57). In (**b**), the effect of *K* versions of the MLP model, representing the number of required normalizations per image to calculate the mean value for each feature, is shown. MAE reduction is also maximized at $K=5$, after which diminishing returns were noted.

In order to determine which of the six candidate regression models was the best predictor, we compared each with the human ratings using Pearson correlation (R, higher is better) and MAE, lower is better). In Fig. 7a the relative success of the six tested regression models is shown. The R values of models are quite comparable, falling between 0.87 and 0.90. However, the MAE of the MLP regressor was much lower when compared to other models (0.57). That is, using the MLP our computer model was able to, on average, predict the human rating of facial normality within 0.57 on a scale of 1–7, and with a correlation of $\hbox {R}=0.9$.

It was observed that the quality of our normalization output was variable due to the stochastic nature of the process. Therefore, we investigated the effect of generating multiple (*K*) versions of $x_{nrm}$ using the K-nearest neighbor algorithm^65^ (Fig. 7b). We began empirically at $K=9$, and measured the $dx_{perc}$ between $x_{raw}$ and each rendition of $x_{nrm}$. For example, when $K=5$ we selected five $x_{nrm}$; i.e., the 5-nearest neighbors of $x_{raw}$ in terms of their perceptual distances $dx_{perc}$. Ultimate feature determination was achieved by averaging the features of these 5-nearest $x_{nrm}$ versions. We observed that increasing *K* up to a value of 5 resulted in a corresponding increase in R. Similarly for MAE, the effect of increasing *K* was beneficial until $K=5$, beyond which diminishing returns were noticed.

**Figure 8:**
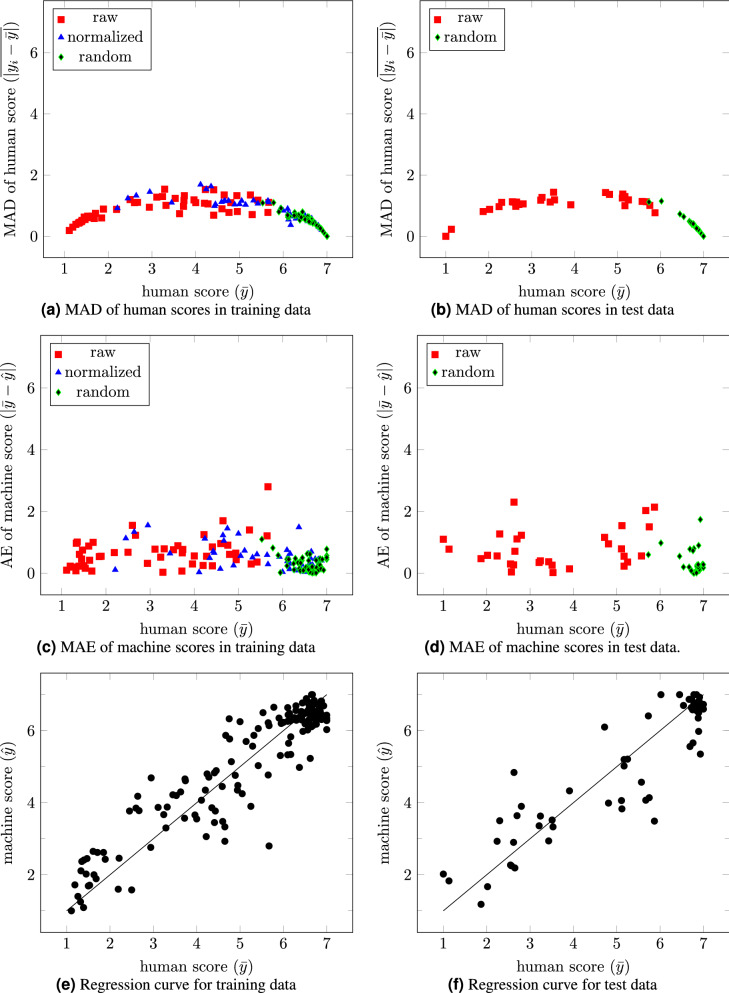
MAD of human facial scoring (**a**,**b**) and MAE of machine relative to human scoring (**c**,**d**) for training and test data. Note average deviation from the mean of human scoring increases for scores in the mid range for both training and testing data (**a**,**b**). On the contrary, machine:human errors are relatively more evenly distributed across the score range. In (**e**,**f**) the relationship of machine to human facial scoring is illustrated for training and test data.

We also investigated the mean absolute deviation (MAD) of human scores for the training and test data. We plotted in Fig. 8a,b the MAD of the mean human score for each image ($\overline{|y_i - {\bar{y}}|}$) versus the mean human score (${\bar{y}}$) for each image in both datasets. It was observed that the statistical dispersion of human scores was relatively less on the extremes of the rating scale (when ${\bar{y}}<3$, MAD was 0.80 for training data and 0.89 for test data; and when ${\bar{y}}>5$, MAD was 0.68 for training data and 0.52 for test data). In contrast, human scores demonstrated greater variance in the mid-range (when $3<{\bar{y}}<5$, MAD was 1.19 for training data, and 1.25 for test data). This finding suggests that the judgment of our human raters was more in agreement for the more normal and abnormal images, while there was slightly more discrepancy in the assessment of the more intermediate faces.

Similarly, we plotted the (MAE) of the machine scores $|{\hat{y}}-{\bar{y}}|$ with respect to human score (${\bar{y}})$ for training and test data in Fig. 8c,d, respectively. Distribution of MAE errors measured 0.77 when ${\bar{y}}<3$, 0.61 when $3<{\bar{y}}<5$, and 0.54 when $3<{\bar{y}}<5$ for training data. In contrast, MAE values were measured as 0.53 when ${\bar{y}}<3$, 0.56 when $3<{\bar{y}}<5$, and 0.56 when $3<{\bar{y}}<5$ for test data. This suggests that our machine scoring paralleled the human scoring across the full spectrum from abnormal to normal images (Fig. 8e,f).

## Discussion

Greater than 100 billion unique human forms have thus far been produced on Earth^66^. While tremendous phenotypic differentiation exists, arguably the most important layer of individuality is one’s distinguishing facial features. With each new recombination, novel variations in facial characteristics such as size, proportion, color, shape, projection, pattern of animation, etc., come forth. This understanding lends some context for the surgeon aiming to reconstruct a deformed face. A facial reconstructive surgeon’s objective is not to transform a face so that it matches a population norm: rather, it is to eliminate anomalies while preserving intact all distinctive facial features unique to a given patient. This objective was mirrored by the success of the first phase of the current project: the development of an automated system to normalize the abnormal components of raw facial images. The subsequent phase of the study was then able to effectively assign a distance measurement between raw and normalized images that closely reflects human evaluation.

In designing a machine learning system to address our stated aims, either a classification or a regression approach could have been undertaken. In a preliminary phase of our work, we tested a classification approach. We collected 2000 internet images representing a wide range of known congenital and acquired facial deformities, and graded them into 5 distinct levels of deformity by visual appraisal. We then trained a convolutional neural network (CNN) to classify the images. What proved problematic to our goal, however, was the enormous variation in age, gender, race, diagnosis, and other characteristics existing within each class. Indeed, faces within each category looked markedly dissimilar from one another. To apply a classification approach to the clinical setting, in which surgeons and their patients place a high premium on the detection of very subtle facial differences, a fine-grained level of measurement is essential. In order to recognize exquisite gradations within a system through the use of a deep learning model, a neural network must be trained using a set of clearly differentiated classes. Thus, our challenge was to assemble a large image database and label it finely on the basis of distinguishing facial characteristics. However, it is difficult for human raters to narrowly classify facial images by features such as race or severity of deformity because the gradations can be indistinct and continuous. In terms of the classification of race, for example, there is inevitable ambiguity due to the historical admixture of human populations. Confidently labeling an individual by skin color, or as Western European versus Central American versus North African, or Central Asian versus Middle Eastern versus Mediterranean, etc., is an unrealistic proposition. Similarly, attempting to construct a broad gradient of facial deformity by human rating of images is problematic. Unless vast numbers of training set images depicting normal and deformed faces of all different types can be obtained - and with a relative distribution matching that of the general population—a CNN will be unable to reliably classify new images contained within a test dataset with the discriminating detail required for the clinical setting. The rarity (some as infrequent as $<1/10^6$ live births) and variety (dozens) of each of the many different facial conditions encountered clinically makes assembling and labeling an adequate image set prohibitive. It is understandable, therefore, that the results of our classification model were poor. For each of the 5 designated classes, the sensitivity (true positive rate) was 0, 0.55, 0.47, 0.48, 0.88; and the specificity (true negative rate) was 1, 0.87, 0.88, 0.87, 0.91, respectively^67^.

Having uncovered the shortcomings of a classification approach, we then attempted a protocol in which we utilized the LPIPS perceptual similarity measure to compare images depicting deformity with an average male and female face (i.e., mean anchors)^68^. Training a computer model to interpret facial images in a manner aligned with human perception, however, represents a major challenge. Superimposed upon the neurophysiologic components of visual perception are multifactorial sources of human subjectivity, and an innate attraction to certain regions of a face more than others^11–14^. Numerous perceptual distance algorithms—LPIPS amongst them—have been introduced in an effort to meet these fundamental challenges^26–29^. We chose to use the recently introduced LPIPS measure because it has been purported to account for some of the nuances of human perception. We tested this distance measure by comparing 25 pairs of pre- and post-reconstructive facial images with their respective gender anchor images. Unfortunately, this approach of measuring the distance between raw images and an average facial standard grossly under-represented the magnitude of difference between pre- and post-operative images relative to the visual interpretation of the investigators. The mean difference between before and after images was only 2.69%. Moreover, post-operative images were labeled as improved (i.e., more normal) only 76% of the time. Our impression was that this method did not provide adequate sensitivity to fine facial changes because the overall digital difference between any given raw image and an average anchor is likely to be extensive. That is, the significance of any particular structural anomaly will be diluted out within the wider backdrop of existing difference between raw and average images. Consider, for example, an average male anchor who appears to be a 30 year old of a given race, when assessing two males with similar modest facial deformities. If one is a 20 year old of the same race as the anchor, and the other an 8 year old of a different race, the comparison may very well be influenced more by race and age than the subtleties of the facial deformity. Therefore, using a normal anchor as a benchmark to expose outlier regions of anatomy is only feasible if it can actually match gender, race, age and all other facial features excluding the area of deformity for every image. This is an unrealistic strategy, as explained above. Furthermore, a similarity measure such as LPIPS is agnostic in how it interprets an image, assessing a landscape of pixels holistically, rather than assigning differential values to certain regions of a face. The significance of anomaly detection and correction using this approach will not be afforded the attention it would otherwise receive from a discriminating human eye.

In considering the study objective further, the process of human facial perception was considered. Within the brain there exists a linked neural network involving various regions in the temporal and occipital lobes that is dedicated to the critical and highly evolved task of interpreting faces^69^. As early as the first few days of life, neonates are able to recognize and mimic faces^70,71^, and the impact of facial perception on social interaction is profound throughout one’s life^72^. Over time, and with a vast experience of visual cognition, humans become extremely adept at recognizing and distinguishing large numbers of faces, and subliminally detecting and evaluating almost imperceptible changes within a face. Humans are also able to instantly imagine the elimination of those differences. In fact, this is the process that a facial reconstructive surgeon goes through during clinical assessment of a patient: detect an anomaly, evaluate the severity, and imagine its correction. This progression is implemented automatically, and in a unique manner, for each individual face encountered.

We determined that a similar approach might enable us to achieve our objective. That is, a comparison protocol between any raw image and it’s *own normalized analogue* (rather than a gender average) might allow for a more discerning extraction of divergent features. This step required the introduction into our protocol of a convolutional neural network face generator (StyleGAN) which delivered a vast potential for creating new faces. The digital data contained in this GAN can be loosely compared to the 20,000 protein-encoding genes in the human genome that permit the formation of an almost boundless number of recombinant human forms^73^. We then designed an algorithm that would iteratively transform any raw facial image—along with information deriving from our generator—into a normalized analogue, by eliminating anomalies while maintaining all unique facial details. By revealing the aberrant regions of the face through the normalization process, we were then able to extract distance features. The normalization step was fundamental to this process; one is unable to define abnormal without establishing a complementary definition of normal. Once discerning the key anomalous features within our raw images, we then trained an MLP model that was able to predict human scores with an average error of 0.57 on a 1–7 Likert scale, and with a Pearson correlation of 0.90 between our machine:human scores. To further validate our model, we then tested our design on the same 25 pairs of pre- and post-operative images that we used in the preceding trial. Our final model performed at a far superior level: mean difference detected between before and after images 48.87% (vs. 2.69%); and post-operative images were labeled as improved (i.e., more normal) 96% of the time (vs. 76%).

As stated above, a facial reconstructive surgeon learns to automatically detect an anomaly, evaluate its severity, and imagine its correction. It can be argued that our model functions so effectively by applying a similar progression, though in a different sequence. First, it normalizes a facial image by filtering out anomalous anatomic features using both a state-of-the-art GAN and an image similarity tool. Then, each raw image is compared to its normalized version using several candidate distance measures from different domains, thereby uncovering and quantifying the anomalous facial elements. Finally, implementation of an optimal regressor integrates raw-normalized distances into a predictive model of human normality ratings.

Certain limitations of our design were noticed. Because our stated goal is to discern granular changes in a face, the system is dependent on the availability of input images of adequate resolution (in general, higher than $256 \times 256$). Image orientation is also critical to the system since the face detection algorithm that we employ hinges on capturing key structural elements in the face (i.e., eyes, mouth, nose). Fortunately, within the clinical setting, images will routinely be of adequate quality to satisfy the model’s requirements. Another possible limitation in our study is that our regression model was trained using human ratings of facial normality. It is appreciated that human appraisal can be influenced by a variety of cognitive biases^74–77^. For example, it is plausible that individuals may rate children’s faces with deformity more favorably than adult faces. Race and gender may also impact human ratings in ways that one would not expect an objective machine system to respond. Thus, with training based on human ratings, we have “built” human bias into our computer model. One could argue that this represents either a limitation or a strength of our design.

The use of the face generator, while fundamental to our study approach, also represents a limitation. StyleGAN was trained on a dataset of “70,000 high-quality PNG images at $1024 \times 1024$ resolution... [containing] considerable variation in terms of age, ethnicity and image background”^25^. While no further details about the dataset are available, its diversity is truly borne out by the ability of our model to consistently and reliably normalize a broad variety of faces across a spectrum of gender, age and racial profiles. We surmise, however, that StyleGAN contains a preponderance of female images because we noted that the computed population average appeared to be female in gender (Fig. 2b). Thus, it was necessary for our design architecture to overcome that bias over the course of the iterative process. Fortunately, it appears to have done so both effectively and reliably (Fig. 4).

Currently, clinical outcomes of surgical interventions are considered primarily through a subjective assessment by patient and surgeon. Over the past decade, there has been increasing attention paid to the development of patient-reported (as opposed to provider-reported) outcome measures (PROMs). While there is no denying the importance of patient/parent attitudes regarding treatment outcome, PROMs are not free of human bias^78,79^, and may not tell the entire story regarding outcome. A PROM doesn’t likely get at the essence of what others may see in a patient’s pre- and post-operative face. Other available outcome measurement techniques that do gauge structural parameters of a face are not benchmarked against a given patient’s own theoretical facial norm, and are not easily adapted for use in the clinical setting. This is where our innovative model promises to fill a crucial gap. We are currently working towards making the technology applicable for handheld smartphone devices. By offering a convenient and truly objective means of measuring both the degree of facial deformity and the extent of improvement achieved through surgical intervention, this approach would represent a fundamental advance in the field through the translation of machine learning to medicine.

## Methods

Nineteen raw facial images were used in this study, all open-source obtained from the internet, and licensed for re-use through the creative commons (all sources included in References). An additional 125 images displayed in this article are newly created transformations of those original 19 images, generated by our novel computer model. A given image $$x_{raw} \in {\mathbb {R}}^{n \times n \times 3}$$ is modeled as superposition of the images $$ x_{nrm}$$ and $$x_{a}$$ where $$x_{raw}$$, $$x_{nrm}$$, and $$x_a$$ denote the raw image, the normalized image, and the anomaly of the given image, respectively. To calculate the anomaly of a given facial image, $$x_a$$ should be quantified and measured objectively by a function. However, it is not possible to directly quantify $$x_a$$ since it is very dependent on $$x_{raw}$$. Further, to the best of our knowledge, there does not exist in the literature a direct metric to put $$x_a$$ on a scale. Short of being able to directly quantify $$x_a$$, we therefore adopted an indirect approach. The initial step of preprocessing involved the detection and alignment of images. Then, we semantically filtered out $$x_a$$ from $$x_{raw}$$ to arrive at $$x_{nrm}$$. After obtaining $$x_{nrm}$$, we then tested a variety of distance measures to measure the difference between $$x_{raw}$$ and $$x_{nrm}$$. This distance reflects the impact of any given facial anomaly, $$x_a$$. As a last step, we built a regression model to predict the extent of facial anomaly represented by the extracted distances in the previous step. These steps are briefly described below.

### Preprocessing

We first detected the face in the given image using dlib’s face detection algorithm^38^. Next, the specific points of the face such as chin, eyes, eyebrows, nose, nostrils, and mouth were detected using dlib’s 68 point landmark detector^38^. Then, we cropped, resized, transformed, and aligned the face by calculating auxiliary vectors from eye to eye and eye to mouth^25^. A face mask was also applied to the aligned image by filling the convex polygon of the outermost points of the face using opencv^39^. At the last step of the preprocessing, the masked area (convex polygon) was enlarged 50 pixels to its border to create some space for the normalization algorithm (refer to Fig. 1). Since StyleGAN produces images of size $1024 \times 1024 \times 3$, the same size was preserved.

### Image normalization

The normalization procedure involved two simultaneous and conflicting objectives working in balance. The first objective was *similarity*, which is measured in ${\mathbb {X}}$ between $x_{raw}$ and $x_{nrm}$, and was responsible for preserving the “normal” features of the individual. The second objective was *averageness*, which was measured in the latent space between the optimization variable *w* and the average vector of the population $w_{avg}$, and was responsible for eliminating the abnormal elements of the facial image. The aim of the normalization procedure is to come up with a candidate latent vector which satisfies both goals. However, every face is unique, and it is practically impossible to build a facial database that could provide these two criteria simultaneously for any given face. Thus, candidate faces should be dynamically generated on the fly.

Generative neural networks such as variational autoencoders (VAEs)^80^ and GANs^81^ are two major generative models which can generate these candidate faces. StyleGAN^25^ is currently the state of the art GAN model, and can generate highly realistic faces in very high resolution. Therefore, we decided to use StyleGAN’s generator as our face source. For any given latent vector $w \in {\mathbb {R}}^{18 \times 512}$, StyleGAN can generate its corresponding face $x \in {\mathbb {R}}^{1024 \times 1024 \times 3}$ by using its generator *G*. To both decrease the complexity of the optimization and to produce realistic images, we used a ${\mathbb {R}}^{1 \times 512}$ latent vector and tiled it 18 times across the layers of generator’s input.

In Algorithm 1 the steps of the proposed normalization method are described. The optimization variable *w* and optimization results $w^*$ are initialized as population average $w_{avg}$, and the best loss value $L^*$ is initialized to infinity. After the initialization phase, loss value *L* and its gradient $\nabla L$ with respect to *w* are calculated and the *w* value is updated at each iteration. Moreover, if the current loss *L* is less than the best loss $L^*$, the best loss $L^*$ and the best latent $w^*$ are saved for later use. The described loop is iterated for *n*=500 times, and $w_{nrm} = w^*$ and corresponding image $x_{nrm} = G(w^*)$ are returned as outputs of the algorithm. We used $L^{S}_{perc}$ as $L^{S}$ and $L^{A}_{mse}$ as $L^{A}$ with constants $\lambda _{sim}=10$ and $\lambda _{avg}=100$, respectively. 
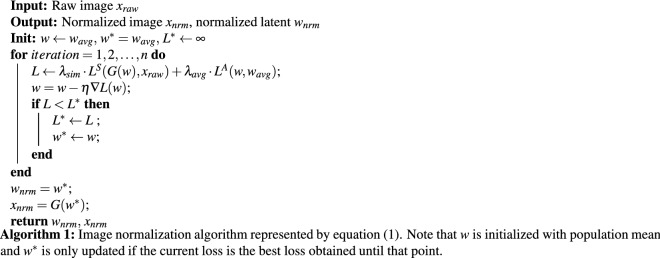


### Feature extraction

A number of different conceptual approaches were utilized in an effort to quantitate a difference between images. Among the most effective were PSNR, MSSIM and LPIPS.

PSNR is a very basic tool to measure the image difference since the images being compared are almost the same except the area which includes the abnormality. PSNR represents the ratio between the maximum possible value of a signal and power of noise (distortion)^28^ and is evaluated as:

2 $$\begin{aligned} dx_{psnr}(x, y) = 20 \log _{10} (\text {MAX}_\text {I}) - 10 \log _{10} \frac{1}{H_l W_l} \sum _{h,w} || ({x}_{hw}, {y}_{hw})||^2, \end{aligned}$$

where *H* and *W* stand for the height and weight of the image, respectively, *h* and *w* denote the index variables along the *H* and *W* directions, and $\hbox {MAX}_\text {I}$ represents the maximum possible value of the pixels. $\text {MAX}_\text {I}$ is set to 255 since each pixel is represented by one byte.

In addition to PSNR, we also employed the MSSIM which quantifies the image degradation between two pairs of images^82^ in a sliding window manner. Given two images *x* and *y*, the SSIM is expressed as:

3 $$\begin{aligned} \text {SSIM}(x, y) = \frac{(2 \mu _x \mu _y + C_1)(2 \sigma _{xy} + C_2)}{ (\mu _x^2 + \mu _y^2 + C_1) (\sigma _x^2 + \sigma _y^2 + C_2)}, \end{aligned}$$

where $\mu _x$ and $\mu _y$ stand for averages of the current sliding window, $\sigma ^2_x$ and $\sigma ^2_y$ denote their variance, $\sigma _{xy}$ is the covariance of *x* and *y*, and $C_1$ and $C_2$ represent some constants. We kept the length of the window, $C_1$ and $C_2$ at their default values, 11, 0.01 and 0.03, respectively. MSSIM is the advanced version of SSIM and incorporates image details at different resolutions obtained by applying low pass filtering and down sampling^27^. Since MSSIM values are limited to [$-1, 1$] and it is employed in our design as a structural distance measurement tool instead of similarity, we used the dissimilarity version of it, namely,

4 $$\begin{aligned} dx_{str}(x, y) = \frac{1- \text {MSSIM}(x,y)}{2}, \end{aligned}$$

which produces the value 0 for exactly same image pairs and 1 for exactly different image pairs.

It has been verified in^26^ that features obtained from the hidden layers of the Deep Convolutional Neural Networks such as SqueezeNet, AlexNet, and VGG are exceptionally good to measure the perceptual difference between two images. Therefore, we employed LPIPS to quantify the distance between two given images. The distance between two images is calculated via:

5 $$\begin{aligned} dx_{perc}(x,y) = \sum _{l} \frac{1}{H_l W_l} \sum _{h,w} || w_l \odot ({\hat{x}}_{hw}^l, {\hat{y}}_{hw}^l)||_2^2, \end{aligned}$$

where *l* denotes the layer of the network, $H_l$ and $W_l$ stand for the height and width of the layer *l*, *h* and *w* represent the current indices of *H* and *W*, $w_l$ contains the optimized weights for layer *l*, and $\odot$ denotes the vector multiplication operation. At each employed layer, the first step is to extract and normalize the features along all pixels. Then each layer is multiplied by a layer specific constant $w_l$. The final score is calculated via $L_2$ distance and summed along *l* layers. In this paper we used the VGG network and its $conv1\_2$, $conv2\_2$, $conv3\_2$, $conv4\_2$ and $conv5\_2$ layers as perceptual measure^83^.

In addition to these three powerful image similarity functions, we also obtained various basic features by comparing images pixel by pixel. Namely, we calculated mean absolute error ($dx_{mae}$), mean squared error ($dx_{mse}$), root mean squared error ($dx_{rmse}$), and logarithm of the hyperbolic cosine error ($dx_{lcosh}$) between $x_{raw}$ and $x_{nrm}$ along their pixels *i* via the Eqs. (6)–(9), respectively^84^, (see Table 1).

**Table 1:** Distance measures employed (left-hand column, with equation numbers) and their key characteristics including metric/semi-metric, algorithmic complexity, noise sensitivity, and efficiency on anomaly detection (right-hand column).

Distance measure	Comments: advantages $(+)$, disadvantages $(-)$
$dx_{psnr}(x, y)$ (refer to Eq. (2), above)	$(+)$ Low complexity, well known technique
	$(-)$ Semi-metric, pixel-wise method, sensitive to noise, inefficient
$dx_{str}(x, y)$ (refer to Eq. (4), above)	$(+)$ Medium complexity, robust to noise, structurally efficient
	$(-)$ Semi-metric, perceptually inefficient
$dx_{perc}(x, y)$ (refer to Eq. (5), above)	$(+)$ Structurally and perceptually efficient, similar to human eye
	$(-)$ Semi-metric, very high complexity
$dx_{mae}({\mathbf {x}}, {\mathbf {y}}) = \frac{\sum _{i} |{\mathbf {x}}_i - {\mathbf {y}}_i|}{n}$ (6)	$(+)$ Metric, very low complexity, better than $dx_{rmse}$
	$(-)$ Pixel-wise method, noise sensitive, inefficient
$dx_{mse}({\mathbf {x}}, {\mathbf {y}})= \frac{\sum _{i} ({\mathbf {x}}_i - {\mathbf {y}}_i)^2}{n}$ (7)	$(+)$ Very low complexity
	$(-)$ Semi-metric, pixel-wise method, highly noise sensitive, inefficient
$dx_{rmse}({\mathbf {x}}, {\mathbf {y}}) = \sqrt{\frac{\sum _{i} ({\mathbf {x}}_i - {\mathbf {y}}_i)^2}{n}}$ (8)	$(+)$ Metric, very low complexity, better than $dx_{mse}$
	$(-)$ Pixel-wise method, noise sensitive, inefficient
$dx_{lcosh}({\mathbf {x}}, {\mathbf {y}}) = \sum _{i} \log (\text {cosh}({\mathbf {x}}_i - {\mathbf {y}}_i))$ (9)	$(+)$ Low complexity, better than $dx_{mae}$, robust to noise
	$(-)$ Semi-metric, pixel-wise method, inefficient
$dw_{man}({\mathbf {u}},{\mathbf {v}}) = \sum _{i}|{\mathbf {u}}_i - {\mathbf {v}}_i|$ (10)	$(+)$ Metric, very low complexity
	$(-)$ Noise sensitive, inefficient
$dw_{euc}({\mathbf {u}},{\mathbf {v}}) = ( \sum _{i} ({\mathbf {u}}_i - {\mathbf {v}}_i)^2)^{1/2}$ (11)	$(+)$ Metric, very low complexity
	$(-)$ Highly noise sensitive, inefficient
$dw_{mink}({\mathbf {u}},{\mathbf {v}}) = ( \sum _{i}|{\mathbf {u}}_i - {\mathbf {v}}_i|^3)^{1/3}$ (12)	$(+)$ Metric, very low complexity
	$(-)$ Highly noise sensitive, inefficient
$dw_{bc}({\mathbf {u}},{\mathbf {v}}) = \frac{\sum _{i} |{\mathbf {u}}_i - {\mathbf {v}}_i|}{\sum _{i} |{\mathbf {u}}_i + {\mathbf {v}}_i|}$ (13)	$(+)$ Very low complexity, robust to noise, efficient
	$(-)$ semi-metric, undefined if $\sum _{i}|{\mathbf {u}}_i + {\mathbf {v}}_i| = 0$
$dw_{can}({\mathbf {u}},{\mathbf {v}}) = \frac{\sum _{i} |{\mathbf {u}}_i - {\mathbf {v}}_i|}{\sum _{i} |{\mathbf {u}}_i| + |{\mathbf {v}}_i|}$ (14)	$(+)$ Metric, very low complexity, robust to noise, efficient
	$(-)$ Undefined if ${\mathbf {u}} = {\mathbf {v}} = 0$
$dw_{che}({\mathbf {u}},{\mathbf {v}}) = \max _{i}|{\mathbf {u}}_i - {\mathbf {v}}_i|$ (15)	$(+)$ Metric, very low complexity
	$(-)$ Highly noise sensitive, inefficient
$dw_{corr}({\mathbf {u}},{\mathbf {v}}) = 1-\frac{({\mathbf {u}}-\bar{{\mathbf {u}}}) ({\mathbf {v}}-\bar{{\mathbf {v}}})}{||{\mathbf {u}}-\bar{{\mathbf {u}}}||_2\ ||{\mathbf {v}}-\bar{{\mathbf {v}}}||_2}$ (16)	$(+)$ Low complexity, robust to noise, better than $dw_{cos}$, efficient
	$(-)$ Undefined if ${\mathbf {u}} = 0$ or ${\mathbf {v}} = 0$
$dw_{cos}({\mathbf {u}}, {\mathbf {v}}) = 1 - \frac{{\mathbf {u}} \cdot {\mathbf {v}}}{||{\mathbf {u}}||_2 ||{\mathbf {v}}||_2}$ (17)	$(+)$ Low complexity, robust to noise, inefficient
	$(-)$ Undefined if ${\mathbf {u}} = 0$ or ${\mathbf {v}} = 0$
$dw_{mah}({\mathbf {u}}, {\mathbf {v}}) = \sqrt{({\mathbf {u}} - {\mathbf {v}})^\top \mathrm{\mathbf {V^{-1}}} ({\mathbf {u}} - {\mathbf {v}})}$ (18)	$(+)$ Low complexity, robust to variation, better than Minkowski family
	$(-)$ Requires variation calculations, inefficient

Note that while images are represented with variables ${\mathbf {x}}$ and ${\mathbf {y}}$, latents are expressed via ${\mathbf {u}}$ and ${\mathbf {v}}$. Similarly, *dx* and *dw* represent distances in image space ${\mathbb {X}}$ and latent space ${\mathbb {W}}$, respectively.

Similar to the image space ${\mathbb {X}}$, we obtained several features in the latent space ${\mathbb {W}}$ by measuring the distance between $w_{raw}$ and $w_{nrm}$. The most basic metrics are the $\hbox {L}_p$ norms for $p= 1$, 2, and 3 labeled with Manhattan (10), Euclidean (11), and Minkowski (12) distances, respectively^62^. In addition to these three basic metrics, we calculated other dissimilarity measures from diversified areas. Bray–Curtis distance (13) is generally used in ecology to compute the population differences of species at two different locations^29^. Canberra distance (14) is another measure mostly used to detect intrusions and to compare ranked lists^58^. Chebyshev distance (15) quantifies the difference between two vectors as the maximum absolute difference between the entries of the two vectors^59^. Correlation distance (16) measures the difference between two vectors as the ratio of their centered dot product to their Euclidean distances. Cosine distance (17) simply measures the angle between the vectors. Mahalanobis distance (18) measures the distance between two vectors extracted from the same distribution by taking into account the correlation between components and rescaling each of them to unit variance^61^. All defining distance measures are tabulated and discussed in Table 1 for easy reference.

### Feature selection

Having extracted a number of possible promising features, we further analyzed the correlation and importance of the features to select the most representative ones to predict human scoring. In this regard, first, the feature correlation matrix was depicted as a heatmap plot to visually assess the extracted features (Fig. 6a). Then, to quantify the relative importance of these features, we applied the ERT^63^ regression model.

As an ensemble method^85^, ERT aims to improve robustness and generalization capability over a single predictor by building numerous base individuals and calculating the average of each predictor as the final output. By averaging the predictions of these individual trees, it reduces the variance of the final prediction and hence improves model’s efficiency and reduces overfitting^86,87^.

The relative importance of a given feature was associated to its depth within the randomized decision tree. Features contributing to the final score prediction for a higher proportion of input data generally reside at the lower level of the tree^88^. Therefore, the relative importance of a feature could be estimated from the expected proportion of samples they contributed to^89^.

After running 1000 ERT, the relative importance of each the 16 features was obtained (Fig. 6b). A 5% relative importance threshold was set as criterion to include a feature for human scoring prediction. As a consequence, the number of features was reduced from 16 to 7. We further removed $dw_{can}$ and $dw_{cos}$ from the selected feature set since they were highly correlated feature pairs ($\ge 0.98$) with $dw_{bc}$ and $dw_{corr}$, respectively.

### Model training and hyper-parameter optimization

Among regressor models, Linear Regressor is the most widely known. It applies the ordinary least squares method to predict a target variable. It can easily be affected by random errors such as outliers in the output variable, and may yield large variance if predictors are correlated with one another. To address this high sensitivity to the target variable, Ridge and Lasso Regressors were introduced in the literature^90^. In addition to the least squares optimization, Ridge and Lasso Regressors manage the variance in the target variable by penalizing the magnitude of the predictor weights via a regularization (shrinkage) parameter $\alpha$. While Ridge Regression applies an $L_2$ regularization onto the predictor weights to minimize the impact of irrelevant features, Lasso regression seeks to minimize the number of nonzero predictor weights through $L_1$ regularization which helps to select only the relevant features. Huber loss function differs from the above-mentioned regression models and assumes a quadratic penalty for small residues and a linear penalty for large residues. Therefore, it decreases the sensitivity to random errors in the target variable and increases robustness^90^. As a regression counterpart of the Support Vector Classifier, Support Vector Regression (SVR) tries to predict the hyperplane fitting the target variable by maximizing the margin and keeping the error within a threshold^91^. Therefore, only the support vectors residing in the margin contribute to the decision boundary and determine the error tolerance of the fitted hyperplane. Although SVR is a non-parametric technique, it is still affected by outliers, because of the possibility of selecting outliers as support vectors. MLP is a feed-forward type artificial neural network consisting of one input layer, one or more hidden layers, and one output layer. MLP can model any linear or nonlinear data by utilizing the nonlinear activation function in its hidden layers and output layer. MLP is trained by using a backpropagation algorithm which iterates backwards the errors from the output layer to the lower layers, and feed-forwards the weight updates from the input layer to the higher layers^92^.

All the implementation was carried out in Python 3.6 using sklearn^89^, scipy^93^, keras^94^ and tensorflow^95^ Python libraries on Intel i9-8950 HK CPU 2.90GHz with NVIDIA GeForce RTX 2070 GPU. The model hyper-parameters were optimized using the tree-structured Parzen estimator (TPE)^96^ algorithm using hyperopt^97^. TPE is a Bayesian sequential model-based optimization which uses previous trials to explore new set parameters in the search space in a tree-like fashion. We first determined the parameters of each regression model and optimized them with a 5-fold cross validation technique on our private dataset. The linear model does not present any hyper-parameter. However, Huber’s hyper-parameter $\epsilon$ was found to be 1.1. SVR’s tree fundamental hyper-parameters were optimized to a linear kernel, $C=0.1$, and $\gamma =0.01$. The $\alpha$ values of the Ridge and Lasso regressors were set to 10.0 and 0.001, respectively^89^. For the MLP model, the hidden layers and neurons were found to consist of only one hidden layer with six neurons. In addition, the activation function was optimized to exponential linear unit for each layer; loss function was found as MSE; and batch size and epoch count were determined to be 20 and 300, respectively^94^.

### Collecting human ratings

**Figure 9:**
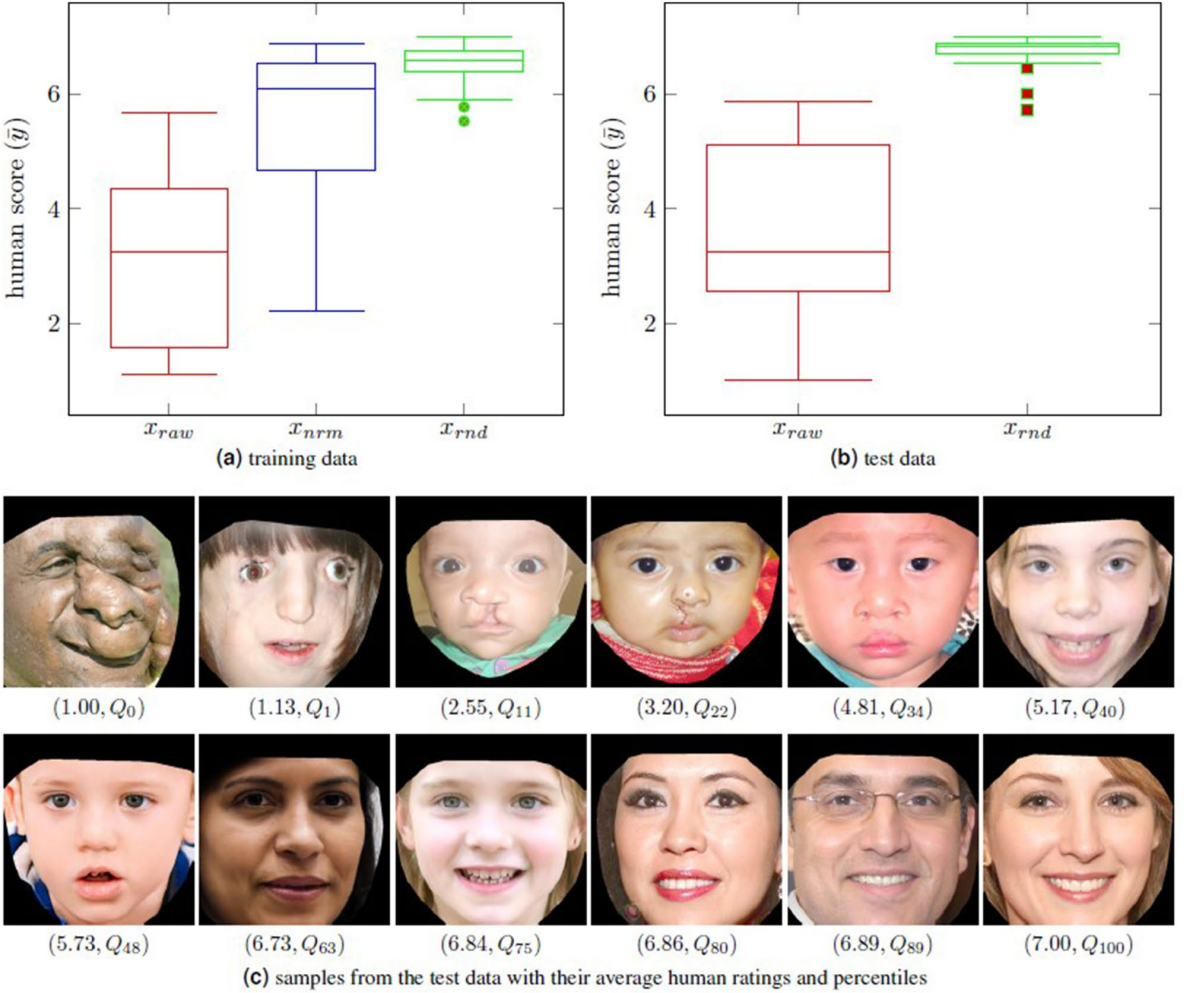
Box and whisker plots of human ratings for the training data (80 raters, 150 images, (**a**) and test data (50 raters, 60 images, (**b**). The first, second (median) and third quartiles ($Q_{25}$, $Q_{50}$, $Q_{75}$) for training data: $x_{raw} = (1.62, 3.27, 4.38)$; $x_{nrm} = (4.69, 6.10, 6.54)$; $x_{rnd} = (6.40, 6.59, 6.77)$. For test data: $x_{raw} = (2.58, 3.34, 5.12)$; $x_{rnd} = (6.74, 6.85, 6.89)$. Note outlier ratings at the lower extremes of both $x_{rnd}$ plots are highlighted. In (**c**) are displayed some representative images from the test data, along with their corresponding mean human ratings and corresponding rating percentiles within the test dataset. Images in the top row are duplicates of raw images exhibited and referenced earlier in this article, and images in the bottom row are transformations created by the study encoder.

The Sidra Medicine Institutional Review Board was consulted prior to publication of this study regarding the collection of human ratings of facial images. Because (1) human raters were recruited from the community at large via word-of-mouth, (2) rating was performed remotely online via password-protected, single-use links, and (3) no personal identifying information was obtained from participants, it was advised that formal consideration of the protocol was not warranted. We obtained the human ratings using two different surveys with distinct faces to create a private training dataset and a public test dataset. For the private training dataset, 80 volunteers aged 18–65 rated 150 images (50 raw with deformities, 50 normalized, 50 randomly generated by the Style GAN). Then, we generated 80 uniquely different surveys, each containing 36 images (12 images from each of the three groups). Raw-normalized image pairs were intentionally placed into different surveys to prevent possible rating bias. For the public dataset, 50 volunteers aged 18–65 rated 60 images (30 raw, 30 random generated by the Style GAN). All images were rated on a 1–7 Likert scale (1 most deformed, 7 most normal). Normality rating distributions are illustrated in Fig. 9a,b for the training and test data, respectively. For the test data, surveyors rated 60 images in five different randomly shuffled orders. Some representative images from the test dataset, along with their corresponding mean human ratings, are shown in Fig. 9c.

## Data Availability

Data supporting the findings of this study are divided into two groups, published data and restricted data. Published data—including open-source images with corresponding normality ratings generated by our model—are available from the corresponding author upon request. Restricted data contains information that could compromise research participant privacy/consent and are not publicly available.
